# Application of Bacteriocins and Protective Cultures in Dairy Food Preservation

**DOI:** 10.3389/fmicb.2018.00594

**Published:** 2018-04-09

**Authors:** Célia C. G. Silva, Sofia P. M. Silva, Susana C. Ribeiro

**Affiliations:** Instituto de Investigação e Tecnologias Agrárias e do Ambiente, Universidade dos Açores, Angra do Heroísmo, Portugal

**Keywords:** bacteriocins, lactic acid bacteria, dairy products, biopreservation, edible coatings

## Abstract

In the last years, consumers are becoming increasingly aware of the human health risk posed by the use of chemical preservatives in foods. In contrast, the increasing demand by the dairy industry to extend shelf-life and prevent spoilage of dairy products has appeal for new preservatives and new methods of conservation. Bacteriocins are antimicrobial peptides, which can be considered as safe since they can be easily degraded by proteolytic enzymes of the mammalian gastrointestinal tract. Also, most bacteriocin producers belong to lactic acid bacteria (LAB), a group that occurs naturally in foods and have a long history of safe use in dairy industry. Since they pose no health risk concerns, bacteriocins, either purified or excreted by bacteriocin producing strains, are a great alternative to the use of chemical preservatives in dairy products. Bacteriocins can be applied to dairy foods on a purified/crude form or as a bacteriocin-producing LAB as a part of fermentation process or as adjuvant culture. A number of applications of bacteriocins and bacteriocin-producing LAB have been reported to successful control pathogens in milk, yogurt, and cheeses. One of the more recent trends consists in the incorporation of bacteriocins, directly as purified or semi-purified form or in incorporation of bacteriocin-producing LAB into bioactive films and coatings, applied directly onto the food surfaces and packaging. This review is focused on recent developments and applications of bacteriocins and bacteriocin-producing LAB for reducing the microbiological spoilage and improve safety of dairy products.

## Introduction

Bacteriocins are generally defined as peptides or proteins ribosomal synthesized by bacteria that inhibit or kill other related or unrelated microorganisms (Leroy and De Vuyst, 2004; Cotter et al., 2005). Bacteriocins may have a narrow spectrum, by inhibiting bacteria taxonomically close, or a broad spectrum, by inhibiting a wide variety of bacteria (Cotter et al., 2005; Mills et al., 2011).

For the past years, bacteriocins have attracted considerable interest for their use as safe food preservatives, as they are easily digested by the human gastrointestinal tract (Mills et al., 2011). The use of bacteriocins as natural food preservatives fulfills consumer demands for high quality and safe foods without the use of chemical preservatives. However, the application of bacteriocins as food additives can be limited for various reasons, such as effectiveness of pathogen elimination or its high price (Chen and Hoover, 2003). Nevertheless, research interest in bacteriocins has continued over the past years, as investigators continuing to search for new and more effective bacteriocins to address both biologic and economic concerns.

The application of bacteriocins for biopreservation of foods usually includes the following approaches: inoculation of food with the bacteriocin-producer strain; addition of purified or semi-purified bacteriocin as food additive; and use of a product previously fermented with a bacteriocin-producing strain as an ingredient in food processing (Chen and Hoover, 2003).

An increasingly number of bacteriocins have been isolated and identified from Gram-positive and Gram-negative microorganisms. As a result, databases have been created to compile the information that can be used for the automated screening of bacteriocin gene clusters (Blin et al., 2013; van Heel et al., 2013).

### Classification of Bacteriocins

For the past years, several classifications of bacteriocins have been proposed taking into consideration the first classification proposed by Klaenhammer (1993). Recently, in order to classify novel bacteriocins, Alvarez-Sieiro et al. (2016) proposed an adjusted classification scheme based on the biosynthesis mechanism and biological activity in accordance with other proposals (Arnison et al., 2013). They propose three major classes: Class I – small post-translationally modified peptides; Class II – unmodified bacteriocins; and Class III – larger peptides (>10 kDa, thermo-labile), being each one subdivided into subclasses.

### Mode of Action

Bacteriocins have distinct mechanisms of action and can be divided into those that promote a bactericidal effect, with or without cell lysis, or bacteriostatic, inhibiting cell growth (da Silva Sabo et al., 2014). Most of the bacteriocins produced from LAB, in particular those inhibiting Gram-positive bacteria, exert their antibacterial effect by targeting the cell envelope-associated mechanisms (Cotter et al., 2013). Several lantibiotics and some class II bacteriocins target Lipid II, an intermediate in the peptidoglycan biosynthesis machinery within the bacterial cell envelope and, by this way they inhibit peptidoglycan synthesis (Breukink and de Kruijff, 2006). Other bacteriocins use Lipid II as a docking molecule to facilitate pore formation resulting in variation of the cytoplasm membrane potential and ultimately, cell death (Machaidze and Seelig, 2003). Nisin, the most studied lantibiotic, is capable of both mechanisms (Cotter et al., 2005). Some bacteriocins damage or kill target cells by binding to the cell envelope-associated mannose phosphotransferase system (Man-PTS) and subsequent formation of pores in the cell membrane (Cotter et al., 2013). Other bacteriocins can kill their target cells by inhibition of gene expression (Parks et al., 2007; Vincent and Morero, 2009) and protein production (Metlitskaya et al., 2006).

## Bacteriocins Produced by Lab

Although there are several microorganisms that produce bacteriocins, those produced by the lactic acid bacteria (LAB) are of particular interest to the dairy industry (Egan et al., 2016). LAB have long been used in a variety of food fermentations by converting lactose to lactic acid, as well as producing additional antimicrobial molecules such as other organic acids, diacetyl, acetoin, hydrogen peroxide, antifungal peptides, and bacteriocins (Egan et al., 2016). As a result of their extensive use in traditional fermented products, most of the LAB are Generally Regarded as Safe (GRAS), granted by the American Food and Drug Agency (FDA). The European Food Safety Authority (EFSA) also granted the Qualified Presumption of Safety (QPS) status to most of the LAB genera, such as *Lactococcus, Lactobacillus, Leuconostoc, Pediococcus*, and some *Streptococcus* (EFSA, 2007). Nevertheless, species of the genus *Enterococcus* and some *Streptococcus* are pathogenic, thus, they do not have GRAS status and were not proposed for QPS status (EFSA, 2007).

Lactic acid bacteria bacteriocins are often active across a range of pH values, resistant to high temperatures and active against a range of food pathogenic and spoilage bacteria (Ahmad et al., 2017). In addition, LAB bacteriocins are sensitive to digestive proteases such as pancreatin complex, trypsin and chymotrypsin, and thus do not impact negatively on the gut microbiota (Egan et al., 2016).

## Application of Purified/Semi-Purified Bacteriocins to Dairy Products

Bacteriocins have been used in the biopreservation of various foods, either alone or in combination with other methods of preservation, known as hurdle technology (De Vuyst and Leroy, 2007; Perez et al., 2014). Although results obtained from culture media may show that bacteriocins inhibit target organisms, the application of bacteriocins into foods must be tested to confirm their effectiveness. Many studies showed the potential of applying bacteriocins or bacteriocin-producing strains into foods, such as meat, dairy products, fish, alcoholic beverages, salads, and fermented vegetables (O’Sullivan et al., 2003; Ramu et al., 2015). To date, only nisin (Nisaplin, Danisco) and pediocin PA1 (Microgard^TM^, ALTA 2431, Quest) have been commercialized as food preservatives (Simha et al., 2012). Although other LAB bacteriocins offered promising perspectives to be used as biopreservatives, as for instance the enterocin AS-48 (Sánchez-Hidalgo et al., 2011) or lacticin 3147 (Suda et al., 2012), no other bacteriocin has been proposed for industrial application. The screening of bacteriocins to be applied to foods requires the fulfillment of some important criteria. Producing strains should be food grade (GRAS or QPS), exhibit a broad spectrum of inhibition, present high specific activity, have no associated health risks, present beneficial effects (e.g., improve safety, quality, and flavor of foods), display heat and pH stability, and optimal solubility and stability for a particular food (Cotter et al., 2005; Leroy and De Vuyst, 2010). Various authors have reported that inactivation of several foodborne pathogens by bacteriocins may differ greatly depending on the food matrix used (Muñoz et al., 2007). Therefore, the effectiveness of different bacteriocins to foodborne pathogens must be tested in all food systems.

Recent applications of bacteriocins into dairy foods to control food-borne pathogens included the inoculation of food with LAB that produce bacteriocins (**Table 1**) or the addition of purified or semi-purified bacteriocins directly to food (**Table 2**). Applying bacteriocin-producing LAB strains as antibacterial starter cultures and protective cultures may confer an advantage over the use of semi-purified/purified bacteriocins. In most cases, bacteriocins are adsorbed into food matrices and are easily degraded, which results in a loss of antibacterial activity. Therefore, an alternative method is the incorporation of bacteriocins into food packaging films/coatings, which improve their activity and stability in complex food systems (Salgado et al., 2015). Many researchers reported the potential of using immobilized bacteriocins in the development of antimicrobial packaging films to control food-borne pathogenic bacteria, such as *L. monocytogenes* (Sánchez-González et al., 2013; Ibarguren et al., 2015; Narsaiah et al., 2015). In fermented foods, the contamination with *L. monocytogenes* is a major concern. Several listeriosis outbreaks have been linked with the consumption of dairy products, in particular soft cheeses, causing a problem to dairy industry and public health authorities (Melo et al., 2015). Despite most dairy products, in particular cheeses, being made from pasteurized milk, contamination with *Listeria* still occurs. Cheeses are ready to use products and are usually conserved at refrigeration temperatures that allow the survival and growth of psychrotrophic bacteria, such as *L. monocytogenes*. Therefore, contamination can occur in later stages of dairy product processing (Carpentier and Cerf, 2011; Melo et al., 2015). Consequently, *Listeria* active bacteriocins emerge as an ideal solution for preventing the growth of this pathogen after cooking or packaging (Cotter et al., 2005). Bacteriocins can also be used to control adventitious non-starter flora, such as non-starter lactic acid bacteria (NSLAB) in cheese and wine, and in this way, contribute to the quality of the final product (Oumer et al., 2001; O’Sullivan et al., 2003). In addition, bacteriocins can also be used to enhance food fermentation, accelerating cheese ripening, and even improve its flavor (Oumer et al., 2001).

**Table 1:** Applications of bacteriocin-producing LAB in dairy products (2000–present).

Bacteriocin-producing strain	Application technique	Product	Features	Reference
*Staphylococcus equorum* WS 2733	Cheese was inoculated with bacteriocin-producing strains	Soft cheese	*L. monocytogenes* growth was completely inhibited at low contamination levels and was reduced by 1-2 log at higher contamination levels.	Carnio et al., 2000
*Lactococcus lactis* IFPL359 (transconjugant)	Starter culture	Goat’s milk cheese	Acceleration of cheese ripening.	Martinez-Cuesta et al., 2001
*Lactococcus lactis* CNRZ481	Adjunct culture	Cheddar cheese	Greater enzyme release through lacticin 481-induced lysis and reduction in the growth of NSLAB by 5 log.	O’Sullivan et al., 2003
*Enterococcus faecalis* A-48-32	Adjunct culture	Skimmed milk and non-fat hard cheese	*Bacillus cereus* was reduced in milk to non-detectable levels after 72 h. In cheese, *B. cereus* decreased progressively, reaching 5.6 log after 30 days.	Muñoz et al., 2004
*Lactococcus* lactis subsp. *lactis* INIA 415	Adjunct culture	Hispánico cheese	Acceleration of cheese ripening.	Avila et al., 2005
*Enterococcus faecium* M241 and M249	Inoculation with bacteriocin-producing strains	Skimmed milk	Co-culture with *E. faecium* showed a delay in the growth *L. monocytogenes* of 6 h and reduced this pathogen by 2 log.	Cocolin et al., 2007
*Lactococcus lactis* CL1 and CL2 (transconjugants, Nis^+^, Ped^+^)	Adjunct to commercial starter culture	Cheese	*L. monocytogenes* was reduced by 2.97 (CL1) and 1.64 log (CL2).	Rodriguez et al., 2005
*Enterococcus faecium* F58	Inoculation with bacteriocin-producing strain	Jben (traditional fresh cheese)	*L. monocytogenes* was reduced to below detection limits after 7 days of storage at 22°C.	Achemchem et al., 2006
*Enterococcus faecalis* A-48-32	Inoculation with bacteriocin-producing strain	Skimmed milk and fresh cheese	Reduced *Staphylococcus aureus*, but efficacy was lower in cheese *as S. aureus* remained 1 log lower than control throughout storage.	Muñoz et al., 2007
*Lactococcus lactis*	Starter culture	Cottage cheese	Reduction of *L. monocytogenes* growth after 2 days, but a re-growth on days 5 and 7.	Dal Bello et al., 2012
*Enterococcus mundtii* CRL35 and *Enterococcus faecium* ST88Ch	Inoculation with bacteriocin-producing strains	Fresh Minas cheese	*E. mundtii* CRL35 displayed a bacteriostatic effect on *L. monocytogenes* up to 12 days at 8°C. *E. faecium* ST88Ch was less effective to control *L. monocytogenes.*	Pingitore et al., 2012
*Lactococcus lactis* L3A21M1 and *Enterococcus faecalis*	Inoculation with bacteriocin-producing strains	Fresh cheese	A combination of strains of *E. faecalis* reduced *L. monocytogenes* by 4 to 5 log after 7 days at 4°C. *Enterococcu*s spp. were more effective in reducing *L. monocytogenes* in cheese.	Coelho et al., 2014
*Enterococcus durans* E204	Adjunct culture	Jben	*L. monocytogenes* throughout the storage period and was undetectable after 6–8 days.	Khay et al., 2014
*Lactococcus lactis* ssp. *lactis CCMM / IAV/ BK1*	Starter culture	Jben	*L. monocytogenes* was reduced to below the detectable level during storage at 7°C from days 1 to 6.	Benkerroum et al., 2002
*Lactobacillus sakei* subsp. *sakei* 2a	Cheese was inoculated with bacteriocin-producing strain	Cheese spread	*L. monocytogenes* was reduced at 4°C and decreased to below detection limit at 15°C, after 22–28 days	Martinez et al., 2015
*Lactobacillus plantarum* CCDM 1078	Inoculation with bacteriocin-producing strains	Cheese and quark based spreads	*L. monocytogenes* was reduced by 1 log in both spreads.	Patrovský et al., 2016
*Staphylococcus equorum* SE3	Inoculation with bacteriocin-producing strains	Cheese model	Inhibition of *L. monocytogenes* growth by 7 log units after 24 h.	Bockelmann et al., 2017
*Enterococcus faecium*	Cheese was inoculated with bacteriocin-producing strains	Fresh whey cheese	*L. monocytogenes* was reduced by 4 log after 6 days of storage at 4°C and was undetectable by the 9th day.	Aspri et al., 2017
*Enterococcus faecium* KE82	Adjunct culture to commercial starter culture	Raw and sterile milk	*L. monocytogenes* was reduced by 4 to 5 log after 72 h at 22°C.	Vandera et al., 2017
*Lactococcus lactis* (nisin producer)	Starter culture	Fresh cheese	*L. monocytogenes* was reduced by 2 log after 7 days of storage at 4°C	Kondrotiene et al., 2018

**Table 2:** Applications of purified/semi-purified bacteriocins to control food-borne pathogens in dairy products (2000–present).

Bacteriocin, producer	Product	Features	Reference
Nisin Z, *Lactococcus lactis* W8	Skim and whole-fat milk	Reduced *L. monocytogenes* (5 log CFU ml^-1^) to undetectable levels in both skim and fat milk within 16 h at 8°C.	Mitra et al., 2011
Nisin Z and A and lacticin 481, *Lactococcus lactis*	Cottage cheese	Cell-free supernatant (CFS) from the bacteriocin producing strains show weak ability to reduce *L. monocytogenes*	Dal Bello et al., 2012
Nisin (commercial preparation, Nisaplin^®^)	Galotyri PDO cheese	Extended the shelf life of fresh Galotyri cheese stored at 4°C by 7 days.	Kykkidou et al., 2007
Nisin A (Nisaplin^®^)	Milk pudding	Nisin A was effective to control spore-forming bacteria and extend shelf life. Reduced heat treatments to improve the flavor and aroma without compromising food safety.	Oshima et al., 2014
Nisin (Nisaplin^®^)	Minas Frescal cheese	Nisin (500 IU⋅mL^-1^) reduced *S. aureus* in curd and whey, but the effect was minor during cheese storage.	Felicio et al., 2015
Nisin, *Lactococcus lactis* N5764	Cow milk	Combined nisin and phenolic compounds have a bacteriostatic effect on *Staph. aureus* and *L. monocytogenes* growth	Alves et al., 2016
Lacticin 3147, *Lactococcus lactis* subsp. *lactis* DPC3147	Yogurt and cottage cheese	Reduced *L. monocytogenes* to undetectable levels in yogurt within 10 min. In cottage cheese reduced 85% of *L. monocytogenes* contamination after 120 min.	Morgan et al., 2001
Lacticin 481, *Lactococcus lactis* L3A21M1	Model fresh cheese	Application of purified lacticin 481 reduced *L. monocytogenes* by 3 log after 3 to 7 days at 4°C.	Ribeiro et al., 2016
Lactococcin BZ, *L. lactis* spp. *lactis* BZ and enterocin KP, *E. faecalis* KP	Skim, half and full fat UHT milks	Lactococcin BZ reduced *L. monocytogenes* in all milks to undetectable levels at 4°C or 20°C. Enterocin KP antilisterial result was affected by fat content.	Yildirim et al., 2016
Leucocin K7, *Leuconostoc mesenteroides* K7	UHT whole-fat milk	Combined leucocin K7 at 80 AU/ml and glycine at 5 mg/ml completely inhibited the growth of *L. monocytogenes* over 7 days	Shi et al., 2016
Reuterin, *Lactobacillus reuteri* INIA PRO 137	UHT skim milk	Combined reuterin and nisin reduced *L. monocytogenes* and *S. aureus* to undetectable levels after 12 days at refrigeration temperatures (4° and 8°C).	Arqués et al., 2011
Variacin, *Kocuria varians* NCC 1482	Dairy food models	Variacin (1% of fermented ingredient) was inhibitory to the growth of *Bacillus cereus* at 8°C, but this inhibition weakened as storage temperature increased.	O’Mahony et al., 2001
Bovicin HC5, *Streptococcus bovis* HC5, and nisin	Minas Frescal cheese	Combined bovicin at and nisin at 600 AU g^-1^ completely inhibited the growth of *L. monocytogenes* after 9 days of storage at 4°C.	Pimentel-Filho et al., 2014
Gassericins A and T, *Lactobacillus gasseri* LA39 and LA158	Custard cream	The combined use of bacteriocins and glycine inhibited the growth of *B. cereus.*	Arakawa et al., 2009
Aureocin A70, *Staphylococcus aureus* A70	UHT skim milk	A partially purified aureocin preparation (16 AU mL^-1^) inhibited *L. monocytogenes* by 5.5 log at 4°C after 7 days.	Fagundes et al., 2016
Enterocin CCM 4231, *Enterococcus faecium* CCM 4231	Saint-Paulin cheese	Reduced *L. monocytogenes* by 4 log, but difference decreased to 1 log after 6 weeks of ripening.	Lauková et al., 2001
Enterocin AS-48, *Enterococcus faecalis* A-48-32	Skimmed milk	Combined with sub-lethal heat treatment (65°C for 5 min) reduced *Staphylococcus aureus* below detection limits.	Muñoz et al., 2007
Ent35-MccV (hybrid bacteriocin), *Escherichia coli* BL21	Skim milk	A complete elimination of *E. coli* was observed after 10 h at 37°C and 4 days at 4°C. *L. monocytogenes* was initially reduced but regrowth after 48 h at 37°C. At refrigeration (4°C), *L. monocytogenes* was reduced by 3 log after 10 days.	Acuña et al., 2015
Enterocins, *Enterococcus faecalis* L3B1K3	Model fresh cheese	Semi purified enterocin (536 μg/g) reduced *L. monocytogenes* to undetectable levels in cheese within 6 h.	Ribeiro et al., 2017

Over 230 bacteriocins produced by LAB have been isolated and reported, but only half of them were identified at the protein or DNA levels (Alvarez-Sieiro et al., 2016). Moreover, a limited number of purified or semi-purified bacteriocins have been tested in food systems, especially in dairy foods.

### Nisin

The bacteriocin nisin is classified as a class-Ia bacteriocin or lantibiotic and is the most characterized and commercially important bacteriocin (Ross et al., 2002). Nisin is licensed as a food preservative (E234) and is recognized to be safe by the Joint Food and Agriculture Organization/World Health Organization (FAO/WHO) Expert Committee on Food Additives (FAO and WHO, 2006; Favaro et al., 2015). To date, eight types of nisin variants were discovered and characterized: nisins A, Z, F, and Q produced by *Lactococcus lactis* and nisins U, U2, P, and H produced by some *Streptococcus* strains (O’Connor et al., 2015). The commercially available form of nisin for use as a food preservative is Nisaplin^TM^, with the active ingredient nisin A (2.5%) and other ingredients such as NaCl and non-fat dry milk (Chen and Hoover, 2003).

Nisin has antimicrobial activity against numerous Gram-positive bacteria, including LAB, pathogens such as *Listeria* and *Staphylococcus*, and the spore forming bacteria, *Bacillus* and *Clostridium* (Chen and Hoover, 2003). One of the earliest applications of nisin was to prevent late blowing in cheese caused by gas-producing *Clostridium* spp. (Galvez et al., 2008).

Nisin has been widely applied in cheese and pasteurized cheese spreads to replace nitrate for preventing the outgrowth of clostridia spores (Abee et al., 1995; Chen and Hoover, 2003). Nisin has also been shown to be effective in the control of different pathogens such as *L. monocytogenes* and *Staphylococcus aureus* in dairy products (Sobrino-López and Martín-Belloso, 2008). In a study of Arqués et al. (2011), nisin was shown to reduce *L. monocytogenes* and *S. aureus* in milk stored at refrigeration temperatures. Several studies also tested the addition of nisin to diverse cheese types (cottage cheese, cheddar, and ricotta-type cheeses) and show an effective reduction of *L. monocytogenes* growth, although limited to 1–3 log cycles (Chen and Hoover, 2003). Several reports indicate that nisin A is not very active against *L. monocytogenes*, but its anti-listerial effect is enhanced by the reduction of pH and addition of NaCl (Chen and Hoover, 2003; Khan and Oh, 2016).

In Minas Frescal cheese, *S. aureus* counts were reduced by approximately 1.5 log cycles after addition of nisin (Felicio et al., 2015). In processed cheese, *Bacillus cereus* and *Bacillus subtilis* were also inhibited by nisin (Sobrino-López and Martín-Belloso, 2008). The use of nisin as an antimicrobial treatment extended the shelf-life of a Greek soft acid–curd cheese (Galotyri) by >21 days (Kykkidou et al., 2007). Incorporation of nisin (at a level of 2.5 mg l^-1^) has also shown to increase shelf-life of Ricotta-type cheese by inhibiting the growth of *L. monocytogenes* for >8 weeks (Davies et al., 1997). Moreover, a high level of retention of nisin was observed over the 10-week storage period, with only 10–32% of nisin loss. Ferreira and Lund (1996) also studied the effect of nisin on survival of the most resistant strains of *L. monocytogenes* in cottage cheese and showed a reduction to approximately 3 log cycles in 3 days. This bacteriocin was also tested in other pasteurized dairy products, such as chilled desserts, flavored milk, clotted cream, and canned evaporated milks, and was shown to reduce post process contaminating bacteria such as *L. monocytogenes* (Galvez et al., 2008). The efficacy of a combination of nisin and bovicin HC5 against *L. monocytogenes* and *S. aureus* in fresh cheese was studied by Pimentel-Filho et al. (2014). They observed a reduction of *L. monocytogenes* to undetected levels after 9 days of storage at 4°C, although the combination of bacteriocins did not prevent the growth of *S. aureus*.

Several studies showed that the antimicrobial activity of nisin is affected by several factors including pH, temperature, composition, structure, and natural microbiota of food (Zhou et al., 2014). Proteolysis in cheese-making process may also affect the activity of nisin and limit its antimicrobial efficacy. Nevertheless, some authors described a limited loss in nisin activity (10–32%) in Ricotta cheese after 10 weeks of storage (Cleveland et al., 2001). Moreover, nisin activity was not affected by proteases in a study on Emmental cheese (Favaro et al., 2015).

The application of nisin in dairy products is also limited to pH values lower than 7, as nisin greatly loses the activity at higher pH (de Arauz et al., 2009). Other studies also mention several limitations on the use of nisin in dairy products, due to its interaction with fat and other components in the food matrix (Favaro et al., 2015). However, the role of fat in the activity of nisin is not entirely clear, as studies on heat-treated cream show the inhibition of *B. cereus* growth by low concentrations of nisin (Nissen et al., 2001). Additionally, nisin was found to prevent spoilage bacteria and extend shelf-life in high-fat milk-based pudding (Oshima et al., 2014). In contrast, homogenization of milk was show to reduce the anti-listerial effects of nisin, demonstrating that the treatment of foods may play an important role in the efficacy of bacteriocins such as nisin (Bhatti et al., 2004).

The wide spectrum of inhibition associated with nisin includes LAB themselves (Abee et al., 1995). Therefore, in dairy foods which require LAB for fermentation processes, the application of nisin presents a great limitation. An alternative consists in employing other bacteriocins with a highly specific activity range (Abee et al., 1995). As a result, there has been a great interest in the search for new bacteriocins with a widespread range of antibacterial activity, stability in different food environments, tolerance to heat, and resistance to proteolytic enzymes.

### Pediocins

Pediocins are a class IIa bacteriocins produced by *Pediococcus* spp. and are commercially available under the name Alta 2341^TM^ or Microgard^TM^ (Garsa et al., 2014). This bacteriocin has been shown to be more effective than nisin against some food-borne pathogens such as *L. monocytogenes* and *S. aureus* (Cintas et al., 1998; Eijsink et al., 1998) and Gram-negative organisms such as *Pseudomonas* and *Escherichia coli* (Jamuna and Jeevaratnam, 2004). The potential application of pediocins to dairy products is further enhanced by its stability in aqueous solutions, its wide pH range, and high resistance to heating or freezing (Sobrino-López and Martín-Belloso, 2008). Despite this high potential, few studies have investigated the addition of pediocins to milk or dairy foods. Pediocin (PA-1) was found to reduce *L. monocytogenes* counts in cottage cheese, cream, and cheese sauce (Pucci et al., 1988). The anti-listerial effect was noticeable over a wide temperature and pH ranges and was particularly effective at low initial *L. monocytogenes* contamination (10^2^ cfu ml^-1^). Recently, Verma et al. (2017) described the production of food-grade pediocin from supplemented cheese whey medium. This semi-purified pediocin containing fermented cheese whey was shown to be effective in reducing *S. aureus* counts and enhancing shelf-life of raw buffalo milk (Verma et al., 2017).

### Lacticins

Lacticins are produced by certain strains of *Lc. lactis* and comprise lacticin 3147 and lacticin 481 (Piard et al., 1992; McAuliffe et al., 1998).

Lacticin 3147 was isolated from an Irish kefir grain used for making buttermilk and is a two-component lantibiotic, requiring both structural proteins to give full biological activity (McAuliffe et al., 1998). This bacteriocin exhibit antimicrobial activity against a wide range of food pathogenic and food spoilage bacteria in addition to other LAB (Sobrino-López and Martín-Belloso, 2008; Martínez-Cuesta et al., 2010). A lacticin 3147 powder preparation was shown to be effective for the control of *Listeria* and *Bacillus* in infant milk formulation, natural yogurt, and cottage cheese (Morgan et al., 2001). However, the high concentrations of lacticin powder used, which represent 10% of product weight, were considered impracticable and non-economic by the authors (Morgan et al., 2001).

Lacticin 481 is a single-peptide lantibiotic that exhibits a medium spectrum of inhibition, mainly active against other LAB, *Clostridium tyrobutyricum* (O’Sullivan et al., 2003) and *L. monocytogenes* (Ribeiro et al., 2016). The use of non-purified lacticin 481 was show to have a mild bacteriostatic activity in milk stored at refrigeration temperatures (Arqués et al., 2011). Yet, the application of semi-purified lacticin 481 to fresh cheeses stored at refrigeration temperatures reduced *L. monocytogenes* by 3 log cycles in 3–7 days (Ribeiro et al., 2016). Nevertheless, the application of lacticins in food systems is not likely to ensure complete elimination of pathogens such as *L. monocytogenes.*

### Enterocins

Enterocins are produced by *Enterococcus* species and comprise a diverse group of bacteriocins, both in terms of their classification and inhibitory spectrum (Egan et al., 2016).

Although most LAB have the GRAS status and can be used safely in food applications, bacteriocinogenic enterococci raise some safety concerns (EFSA, 2007). Enterococci are among the pathogens that have been associated with a number of infections in humans (Moreno et al., 2006). In addition, some enterococci may harbor virulence determinants and antibiotic resistance genes (EFSA, 2007). While food enterococci have fewer virulence determinants than clinical strains, they are known for their capacity to exchange genetic information (Eaton and Gasson, 2001). Considering the safe concerns of using bacteriocinogenic enterococci, the use of purified enterocins may be considered more suitable for food consumption.

Enterocin AS-48 is produced by *Enterococcus faecalis* and is a class IIc cyclic bacteriocin that is active against a number of *Bacillus* and *Clostridium* sp. (Egan et al., 2016). This is one of most studied bacteriocins, showing high stability to pH and heat, which makes a great candidate for application in food. Muñoz et al. (2007) tested the efficacy of the enterocin AS-48 for controlling staphylococci in skimmed milk. They observed a bactericidal effect proportional to the bacteriocin concentration (10–50 μg ml^-1^), but complete elimination of staphylococci was not achieved for any of the concentrations tested. This apparently lower effectiveness of AS-48 in milk when compared to culture medium could be attributed to a higher retention of the bacteriocin molecules by milk components and slower diffusion (Muñoz et al., 2007).

Yildirim et al. (2016) investigated the antimicrobial effects of enterocin KP toward *L. monocytogenes* in skim, half fat, and full fat milks. Enterocin KP had a high anti-listerial effect, but this bactericidal effect decreased as both the fat content of milk and inoculation amount of *L. monocytogenes* increased. Also, Lauková and Czikková (1999) reported an inhibitory effect of purified enterocin CCM 4231 (3200 AU ml^-1^) on the growth of *S. aureus* and *L. monocytogene*s in skimmed milk and yogurt. Still, the antagonistic effect of enterocin on *S. aureus* in yogurt was less influential after 24 h.

Usually, enterocins are active against foodborne pathogens, such as *Listeria* spp. and *Clostridium* spp., but their application in food systems is not likely to prevent the re-growth of pathogens throughout the storage time (Zacharof and Lovitt, 2012; de Souza Barbosa et al., 2015). However, Arqués et al. (2011) observed the reduction of *L. monocytogenes* counts in milk below the detection limit after 4 and 24 h by the combined effect of two bacteriocins, reuterin and enterocin AS-48.

Recently, Ribeiro et al. (2017) reported the efficacy of a semi-purified enterocin produced by an *E. faecalis* strain, in reducing the contamination of *L. monocytogenes* in fresh cheese in a dose-dependent manner. Moreover, the highest dose applied to cheeses (approximately 2000 AU g^-1^ of cheese) resulted in reduction of this pathogen below detection levels and this effect remained for all the storage time (72 h).

### Other Bacteriocins

Lactococcin BZ is produced by some strains of *Lc. lactis* spp. (lactis BZ) and has a wide antibacterial activity against Gram-positive and Gram-negative bacteria (Şahingil et al., 2011). This bacteriocin also displayed strong anti-listerial activity in milk. The partially purified lactococcin BZ (400–2500 AU ml^-1^) reduced *L. monocytogenes* counts to an undetectable level in both skim and full-fat milk during storage at 4 and 20°C (Yildirim et al., 2016). In addition, this anti-listerial activity was stable until the end of the storage period (25 days) and was not adversely affected by milk fat content.

Aureocin A70 is a class II bacteriocin produced by a *S. aureus* strain isolated from pasteurized commercial milk (Fagundes et al., 2016). This bacteriocin exerted a bactericidal effect on a wide range of Gram-positive bacteria, including *L. monocytogenes*, and was tested in UHT-treated skimmed milk by Fagundes et al. (2016). Aureocin A70 caused a time-dependent reduction in *L. monocytogenes* counts (5.51 log units) up to 7 days of incubation, but was insufficient to achieve the total elimination of the pathogen, resulting in <0.001% survivors (Fagundes et al., 2016).

Genetically engineered bacteriocins have been proposed to overcome the narrow range of activity of most bacteriocins. Acuña et al. (2012) reported the construction of a chimerical bacteriocin named Ent35-MccV. This hybrid bacteriocin combines in a single molecule the anti-listerial activity of enterocin CRL35 and the anti-*E. coli* activity of microcin V. This hybrid wide-spectrum bacteriocin was active against pathogenic strains of *L. monocytogenes* and *E. coli* O157:H7 and was effective in controlling the growth of both pathogens in skim milk (Acuña et al., 2015).

## Application of Bacteriocin-Producing Bacteria to Dairy Products

Despite the recent advances in bacteriocin research for food applications, the use of purified bacteriocins in the dairy industry remains limited. Frequently, the application of a bacteriocin alone does not provide sufficient protection against microbial contamination of dairy products. The high cost of bacteriocin isolation and purification also limits the commercial exploration of new bacteriocins. In addition, the restrictive food legislation of the health regulatory authorities (FDA and EFSA) limits the approval of new bacteriocins as food preservatives and, as a consequence, only two bacteriocins (nisin and pediocin) are currently commercially available.

The use of bacteriocin-producing bacteria to control contamination microorganisms is an alternative for the use of purified bacteriocins as food additives. Many LAB genera and species have a long history of apparent safe use and they have been granted the GRAS and QPS status. In this regard, incorporation of such bacteria into foods offers a viable solution for controlling contamination microorganisms (**Table 2**). In addition, LAB are commonly used as starter cultures in food fermentations. Thus, researchers have explored the *in situ* production of bacteriocins by adding protective cultures that may grow and produce bacteriocins during the manufacture and storage of dairy foods. Many studies have also focused on the selection and development of bacteriocinogenic cultures as cell lysis-inducing agents to improve cheese maturation and flavor (Beshkova and Frengova, 2012). In addition, the use of bacteriocin-producing LAB has been proposed to prevent late blowing occurring in cheeses. Late blowing defect is a major cause of spoilage in ripened cheeses, resulting in the appearance of texture and flavor defects, due to the ubiquitous presence of *Clostridium* spores (Gómez-Torres et al., 2015). The most common strategies to reduce *Clostridium* spores are often not sufficient to prevent late blowing in cheeses (Garde et al., 2011) and the use of bacteriocinogenic LAB emerges as an alternative strategy (**Table 3**).

**Table 3:** Applications of bacteriocin-producing LAB to prevent gas blowing in cheese caused by *Clostridium* spp. (2000–present).

Bacteriocin-producing strain	Application technique	Product	Features	Reference
*Lactobacillus plantarum* TF711	Adjunct to commercial starter culture	Cow milk cheese	Reduction of 2⋅2 log units of *Clostridium* spores.	González and Zárate, 2015
*Lactobacillus reuteri* INIA P572	Adjunct to commercial starter culture together with glycerol (required for reuterin production).	Cow milk cheese	Controlled the growth of *Clostridium* and prevented the development of late blowing.	Gómez-Torres et al., 2014
*Lactobacillus gasseri* K7	Adjunct to commercial starter culture	Semi-hard cheese	Failed to reduced *Clostridium* spores, but delayed blowing of cheeses.	Matijasic et al., 2007
*Lactococcus lactis* IFPL 3593	Adjunct to commercial starter culture	Semi-hard cheeses	5 log g^-1^ reduction in the numbers of spores.	Martínez-Cuesta et al., 2010
*Lactococcus lactis* ssp. *lactis* IPLA 729	Adjunct to starter culture	Vidiago cheese	Reduced *C. tyrobutyricum* by 3 log during ripening	Rilla et al., 2003
*Lactococcus lactis* subsp. *lactis* INIA 415	Starter culture	Ovine milk cheese	Outgrowth inhibition of *Clostridium* spores without altering sensory characteristics.	Garde et al., 2011
*Streptococcus macedonicus* ACA-DC 198	Adjunct to starter culture	Kasseri cheese	Reduced *C. tyrobutyricum* by 1.4 log during ripening	Anastasiou et al., 2009
*Streptococcus thermophilus* 580	Starter culture	Milk curd	No gas production for up to 20 days.	Mathot et al., 2003

Among the various LAB species/strains producing bacteriocins, *Lactococcus* sp. has gained a particular interest in the biopreservation of dairy foods. Benkerroum et al. (2002) tested the effect of *in situ* bacteriocin production of *L. lactis* ssp. *lactis* against *L. monocytogenes*, in a traditional fermented milk (lben). They found that *L. monocytogenes* decreased to below the detectable level within 24 h of storage at 7°C in lben fermented with the bacteriogenic starter culture. Moreover, the pathogen was efficiently inactivated from contaminated samples despite the high level of contamination (10^7^ cfu ml^-1^) for up to 6 days of storage at 7°C (Benkerroum et al., 2002).

The application of nisin-producing *Lactococcus* sp. in dairy foods which requires LAB starters presents a problem, because the wide spectrum of inhibition associated with nisin includes LAB themselves (Abee et al., 1995). Still, Yamauchi et al. (1996) produced a yogurt by incorporating a nisin-producing strain, *L. lactis* subsp. *lactis*, in raw milk. The bacteriocin-producing LAB were killed before the addition of the traditional yogurt cultures, and this resulted in increased storage life of the yogurts by preventing the growth of spoilage bacteria (Yamauchi et al., 1996).

Recently, Kondrotiene et al. (2018) reported a reduction in *L. monocytogenes* when three nisin A producing *Lc. lactis* strains were added to fresh cheese. However, the reduction on *Listeria* contamination was limited to 2 log units within 7 days of cheese storage. Likewise, the use of a nisin A-producing *Lactococcus diacetylactis* in a mixed starter culture was not effective in inhibiting *L. innocua* growth in Cheddar cheese (Benech et al., 2002). Maisnier-Patin et al. (1992) examined the inhibitory effect of another nisin-producing *Lc. lactis* on *L. monocytogenes* in Camembert cheese. In the presence of nisin-producing starter, the numbers of the pathogen decreased until the end of the second week, leading to a reduction of 3 log cfu g^-1^, but a regrowth of *Listeria* was observed in cheeses throughout ripening (6 weeks).

A reduction of *L. monocytogenes* counts was observed in raw milk cheese by the use of two nisin-producing *Lactobacillus lactis* subsp. *lactis* as starter cultures (Rodrígez et al., 2001). In contrast, the use of nisin-producing strains to control *E. coli* and *L. monocytogenes* in Feta and Camembert cheeses has shown limited results (Ramsaran et al., 1998). *E. coli* O157:H7 survived the manufacturing process of both cheeses and was present in cheese made with nisin-producing strains at the end of 75 days of storage in greater numbers than the initial inoculum. The Feta cheese that contained nisin was the only cheese in which *L. monocytogenes* remained at the level of the initial inoculum after 75 days of storage. The use of bacteriocinogenic pediococci in dairy products also exhibited limited results due to their inability to ferment lactose (Renye et al., 2011).

Mills et al. (2011) tested a plantaricin-producing *Lactobacillus plantarum* strain as an anti-listerial adjunct in the presence and absence of nisin-producing starters for the manufacture of cheeses. The combination of *Lb. plantarum* strain (at 10^8^ cfu ml^-1^) with a nisin producer reduced *Listeria* to undetectable levels by day 28. Moreover, they found that *Lb. plantarum* was much more effective at inhibiting *Listeria* than the nisin producer alone.

Carnio et al. (2000) studied the anti-listerial potential of a food-grade strain *Staphylococcus equorum*, producing a bacteriocin named as micrococcin P1, in soft cheese. A remarkable reduction of *L. monocytogenes* growth was achieved, but this effect was dependent on the contamination level. In addition, a regrowth of the viable *Listeria* could be observed after 10–16 days of maturation. Likewise, Dal Bello et al. (2012) detected a reduction of *L. monocytogenes* by 3 log units in cottage cheese, after incubation with a lacticin-481-producing *Lc. lactis* strain. However, after an initial period, *L. monocytogenes* counts increased to values comparable to the control.

In addition to natural bacteriocinogenic strains, the heterologous production of bacteriocin by genetically engineered LAB was tested in dairy foods (Leroy and De Vuyst, 2004). A lacticin 3147 transconjugant generated via conjugation of bacteriocin-encoding plasmids was used successfully against *L. monocytogenes* in cottage cheese (McAuliffe et al., 1999). Reductions of 99.9% were seen in cottage cheese held at 4°C after 5 days. In another study, a lacticin 3147 transconjugant presented also a protective effect when applied to the cheese surface, reducing *L. monocytogenes* by 3 log units (Ross et al., 2000). In contrast, this protective effect was not evident when a nisin-producing culture was used, possibly due to pH instability (Ross et al., 1999).

The use of *Enterococcus* spp. in foods may represent a risk for consumers and requires a safety assessment by the European food authorities (EFSA, 2007). However, enterococci are found naturally in some dairy foods, as they are used as starter cultures and are often part of the microbiota of artisanal cheeses (Domingos-Lopes et al., 2017). In addition, some strains of bacteriocinogenic enterococci were show to lack many of the virulence determinants (Jaouani et al., 2015). De Vuyst et al. (2003) suggested that *Enterococcus* species could be safely used in food if virulence genes were absent. Consequently, several studies have also employed enterocin-producing enterococcus in food systems.

The inhibitory effect of enterocin-producing enterococci against *L. monocytogenes* and *S. aureus* in dairy foods such as milk and cheeses was demonstrated by several authors (Giraffa, 1995; Giraffa and Carminati, 1997; Nunez et al., 1997; Lauková et al., 1999a,b: Lauková et al., 2001). Many enterococcal strains producing enterocins were isolated and found to be effective in controlling contamination in cheeses, without compromising the acid-producing activity of the starter and the organoleptic characteristics of the final product (Khan et al., 2010). Bacteriocinogenic *E. faecalis* strain was show to reduce *L. monocytogenes* counts in Manchego cheese by 1 and 2 log units after 7 and 60 days, respectively (Nunez et al., 1997). Although inoculation of milk with *E. faecalis* strain reduced the rate of acid production in the curd, the flavor and bitterness of the final product were not influenced.

Other bacteriocinogenic enterococci have also been reported to reduce *L. monocytogenes* in dairy foods. Pingitore et al. (2012) investigated two bacteriocinogenic enterococci strains (*Enterococcus mundtii* CRL35 and *Enterococcus faecium* ST88Ch) isolated from cheeses. Growth of *L. monocytogenes* was inhibited in Minas cheeses containing *E. mundtii* up to 12 days of storage at 8°C. This bacterium displayed a bacteriostatic effect since *Listeria* counts remained similar to the initial inoculum. *E. faecium* strain was found to be less effective, as the bacteriostatic affect occurred only after 6 days at 8°C.

Further studies also demonstrated the inhibitory effect of enterocin-producing enterococci strains against *L. monocytogenes* in raw milk. Vandera et al. (2017) investigated the use of a multiple enterocin-producing bacterial strains possessing the structural entA, entB, and entP enterocin genes. Some strains exhibited a bacteriostatic effect on *L. monocytogenes* in raw milk incubated at 37°C for 6 h. When raw milk cultures were further incubated at 18°C, viable populations of the pathogen were reduced slightly (by 0.2–0.4 log cfu ml^-1^) after 24 h and up to 72 h.

Achemchem et al. (2006) studied the effectiveness of an *E. faecium* strain in controlling *L. monocytogenes* in goat’s milk and goat’s traditional cheese (jben). Coculture experiments of *E. faecium* and *L. monocytogenes* in milk demonstrated that the pathogen was not eliminated, but when the bacteriocinogenic strain was previously inoculated in whole milk and left to grow for 12 h before contamination, *Listeria* was undetectable after 130 h of coculture. Moreover, addition of the bacteriocinogenic strain to jben cheese contaminated with *L. monocytogenes* prior to packaging, reduced the number of viable *Listeria* to undetectable levels, after 1 week of storage at 22°C.

Coelho et al. (2014) examined the inhibitory effect of enterocin-producing *E. faecalis* strains against *L. monocytogenes* in fresh cheese. Inoculation of milk with bacteriocinogenic *E. faecalis* strains was shown to reduce *L. monocytogenes* counts by 3–4 log units in fresh cheese compared to the control. The combination of two enterocin producers optimized the reduction of *Listeria* counts in fresh cheese, decreasing this pathogen by 4 log cfu g^-1^ in the first 3 days of storage and by 5 log cfu g^-1^ on day 7.

Bacteriocin-producing LAB strains have also been assessed to improve cheese maturation and flavor. These LAB cultures may induce controlled lysis of starter and/or non-starter LAB (NSLAB) and subsequent intracellular release of proteinases and peptidases, resulting in rapid onset of proteolysis and cheese ripening. *Lc. lactis* producing lacticin 3147 was shown to accelerate cheese ripening and also prevent late blowing in cheese by the inhibition of clostridia growth (Martínez-Cuesta et al., 2010).

Various bacteriocin-producing strains were shown to possess a lytic effect on starter cultures. The use of bacteriocin-producing *Lc. lactis* ssp. *cremoris* as a starter adjunct in Cheddar cheese manufacture was show to increase the rate of starter lysis. Cheese manufactured with the bacteriocinogenic adjunct exhibited increased cell lysis and higher concentrations of free amino acids, with associated higher sensory evaluation scores (Morgan et al., 1997). Another bacteriocin-producing starter *Lc. lactis* (a lacticin 3147 producer) was tested for controlling the proliferation of undesirable microorganisms during cheese manufacture. Cheeses made with lacticin 3147-producing starters exhibited significantly lower levels of NSLAB that remained constant over 6 months of ripening (Ryan et al., 1996).

Some strains of lacticin 481-producing *Lc*. *lactis* were also tested in cheese production and showed to cause partial lysis of starter lactococcal cultures, which continue to grow at a slower rate (O’Sullivan et al., 2003). As a direct result of starter lysis with concomitant enzyme release in the cheese matrix, these strains may be employed to increase cheese ripening. Additionally, a three-strain starter system was tested on cheese by Morgan et al. (2002). This system comprised a bacteriocin producer which causes the lysis of a second strain (sensitive to bacteriocin) and a third strain resistant to bacteriocin activity, for acid production during cheese manufacture. The experimental cheese made with this three-strain starter system showed an increase in lysis and decrease in bitterness compared to cheeses manufactured without the bacteriocin-producing adjunct (Morgan et al., 2002).

## Combining Bacteriocins With Other Hurdles

One of the approaches to improve the protective action of bacteriocins is the combination with other hurdles such as chemical additives (such as EDTA, sodium lactate, potassium diacetate, and others), heating, and high-pressure treatments (Egan et al., 2016).

Narayanan and Ramana (2013) observed that the use of pediocin in combination with eugenol incorporated into polyhydroxybutyrate films worked in synergized form and provided an effective hurdle preventing food contamination. Other researchers used successfully the mixture of bacteriocins and EDTA in the sensitization of Gram-negative bacteria (Prudêncio et al., 2015). Gram-negative bacteria become sensitive to bacteriocins if the permeability of their outer membrane is compromised with chelating agents, such as EDTA (Chen and Hoover, 2003). Zapico et al. (1998) demonstrated a synergistic effect of the combined use of nisin and the lactoperoxidase system (LPS) to control *L. monocytogenes* in skim milk. A listericidal effect (5.6 log units lower than the control milk) was observed in treatments containing nisin (10 or 100 IU ml^-1^) with LPS, after 24 h at 30°C. Moreover, when the two preservatives were added in two steps (LPS was added after 3 h and nisin after 5 h of growth), the difference in *L. monocytogenes* counts increased by 7.4 log units.

Several authors also observed the synergistic effect of bacteriocins after temperature treatments (Prudêncio et al., 2015). Kalchayanand et al. (1992) exposed bacteriocin-resistant bacteria to sub-lethal stresses, either at low or high temperatures, and treated with nisin and pediocin. They found that both bacteriocins were effective in reducing the cell viability. This synergistic effect was also observed in Gram-negative bacteria, which are normally insensitive to these bacteriocins (Boziaris et al., 1998).

High pressure processing is a common technique for inactivating microorganisms at room temperature, but this treatment does not ensure the complete inactivation of microorganisms (Prudêncio et al., 2015). Several studies have demonstrated the synergistic effect of bacteriocins such as nisin with high pressure processing on the inactivation of food microorganisms (Garriga et al., 2002; Zhao et al., 2013). It is well documented that the use of bacteriocins in combination with these processing techniques enhances bacterial inactivation (Chen and Hoover, 2003). As an example, Rodriguez et al. (2005) demonstrated the efficacy of the application of reduced pressures combined with bacteriocin-producing LAB to improve cheese safety.

## Incorporation of Bacteriocins in Antimicrobial Films and Coatings

A common strategy for preservation of foods that are eaten raw or without further cooking is the application of edible films or coatings containing antimicrobial substances. The incorporation of antimicrobial compounds such as bacteriocins in edible coatings and films presents as an interesting alternative for ensuring the control of pathogenic microorganisms in food products (Valdés et al., 2017).

Edible coatings and films are composed of thin layers of biopolymers that modify the surrounding atmosphere of foods, forming a barrier between the food and the environment, improve the safety, quality, and functionality of food products without changing organoleptic and nutritional properties (Han, 2003; Valdés et al., 2017). The use of purified bacteriocins or bacteriocin-producing bacteria in the packaging system may be more effective in the inhibition of the growth of pathogenic and/or deterioration microorganisms throughout the extent of the latency phase (Balciunas et al., 2013). Hydrocolloids (proteins and polysaccharides) are the most extensively investigated biopolymers in edible coatings and films applied to cheese. They facilitate the incorporation of functional compounds such as bacteriocins and bacteriocin-producing bacteria and allow an increase in the stability, safety, and shelf-life of dairy foods (Scannell et al., 2000a).

To date, few studies have investigated the effectiveness of incorporating bacteriocins and/or bacteriocin-producing LAB in coatings and films applied to dairy products. Some authors observed an inhibition of the growth of pathogenic microorganisms in foods packed with coatings and films containing antimicrobial metabolites synthesized by LAB (Cao-Hoang et al., 2010; da Silva Malheiros et al., 2010; Ercolini et al., 2010; Aguayo et al., 2016; Malheiros et al., 2016) or containing viable LAB in the film/coating matrix (Concha-Meyer et al., 2011; Barbosa et al., 2015).

Studies of the effectiveness of incorporating purified bacteriocins in edible coatings show a limited reduction of pathogens such as *L. monocytogenes*. Cheeses, particularly fresh cheeses, are highly perishable due to their high content in caseins, lipids, and water. The complexity of cheese composition and its manufacture support the development of pathogenic and deteriorating microorganisms that increase the risk of foodborne illness and reduce cheese quality and acceptability (Ramos et al., 2012). By acting as additional hurdle, the application of edible coatings and films with incorporation of bacteriocins may overcome problems associated with post-process contamination, therefore enhancing the safety and extending the shelf-life of the cheese.

Cao-Hoang et al. (2010) incorporated nisin in films of sodium caseinate applied in semi-soft cheese and observed a small reduction in *L. innocua* counts (1.1 log cfu g^-1^) after a week of storage at 4°C. In another study, the incorporation of nisin and lacticin in cellulose coatings applied to Cheddar cheese reduced levels of *L. innocua* by 2 log cycles, and *S. aureus* by 1.5 log cycles (Scannell et al., 2000b). However, other studies have shown an effective reduction on pathogen growth. In Ricotta cheese coated with galactomannan and nisin, the growth of *L. monocytogenes* was prevented for 7 days at 4°C (Martins et al., 2010). The application of a coating in Port Salut cheese, consisting of tapioca starch combined with nisin and natamycin, reduced *L. innocua* counts above 10 cfu ml^-1^ during storage, acting as a barrier to post-process contamination (Resa et al., 2014).

Recently, Marques et al. (2017) used a biodegradable film incorporated with cell-free supernatant (CFS) containing bacteriocin-like substances of *Lactobacillus curvatus* P99, to control the growth of *L. monocytogenes* in sliced “Prato” cheese. These films containing the bactericidal concentration of CFS were able to control *L. monocytogenes* for 10 days of storage at 4°C.

## Conclusion

This review highlights the most recent trends in the use of bacteriocins and bacteriocin-producer bacteria in dairy foods. Bacteriocins, either *per se* or produced by live bacteria, can be successfully incorporated into dairy products to assure safety, extending shelf-life and preserve quality. The use of purified and concentrated bacteriocins as food additives has been the preferred method due to more efficacy, compared to direct application of bacteriocinogenic cultures. However, the effectiveness of bacteriocins in food systems is often low due to several factors such as adsorption to food components, enzymatic degradation, poor solubility, or uneven distribution in the food matrix. On the other hand, the application of live bacteriocin-producing bacteria into dairy foods may overcome the limitations on the use of purified bacteriocins. The bacteriocinogenic LAB added to dairy products such as yogurts and cheeses will ensure continuous production of bacteriocins throughout maturation and storage, and may be included as starter/adjunct cultures in fermentation. The main difficulty associated with this application is the lack of compatibility between the bacteriocin-producing strain and other cultures required in the fermentation of dairy foods.

Studies on application of bacteriocins and/or bacteriocin producers have been focused mainly on cheeses because most discovered LAB bacteriocins were effective against *Listeria monocytogenes*. This foodborne pathogen is often a great concern in traditional cheeses made from raw milk and as a post-processing contaminant in cheeses made from pasteurized milk. Cheeses are particularly vulnerable to the contamination of *L. monocytogenes* because they provide the appropriate growth conditions for this pathogen. Listeriosis out-breaks linked to the consumption of contaminated cheeses have been reported worldwide. Therefore, the application of bacteriocins as natural preservatives for improving cheese safety has attracted significant research interest in recent years. In contrast, very limited research has been focused on the application of bacteriocins to the preservation of milk, cream, yogurts, and other dairy products.

Although most efforts have been devoted to discover novel bacteriocins with unique properties, more studies are necessary for effective application of bacteriocins in dairy foods in order to understand bacteriocin performance in the complex environment of food matrices. In addition, bacteriocins may be combined with other protection tools as part of hurdle technologies to ensure bio-preservation and shelf-life extension of dairy foods. Their use may lie in the combination with other preservation techniques, or in the incorporation in biofilms and active packaging. In the latter case, further studies are necessary to ensure the adaptation of edible coatings and films to bacteriocin activity and efficacy in dairy products.

## Author Contributions

All authors listed have made a substantial, direct and intellectual contribution to the work, and approved it for publication.

## Conflict of Interest Statement

The authors declare that the research was conducted in the absence of any commercial or financial relationships that could be construed as a potential conflict of interest.
